# Monitoring of drug treatment and psychosocial intervention with SPECT in Alzheimer patients Implications for neurologically appropriate psychosocial interventions. An observational study. The Osaki-Tajiri Project

**DOI:** 10.1590/1980-57642018dn12-040007

**Published:** 2018

**Authors:** Kenichi Meguro, Shigeo Kinomura, Kenji Sugamata, Tachio Sato, Keiichi Kumai, Junko Takada, Satoshi Yamaguchi

**Affiliations:** 1 Geriatric Behavioral Neurology, Tohoku University CYRIC, Japan; 2 Clinic of Imaging Medicine and Brain Health, Japan; 3 The Osaki-Tajiri SKIP Center, Japan.

**Keywords:** Alzheimer’s disease, donepezil, psychosocial intervention, monitoring, cerebral blood flow, eZIS, SPECT, doença de Alzheimer, donepezila, intervenção psicossocial, monitorização, fluxo sanguíneo cerebral, eZIS, SPECT

## Abstract

We previously examined cerebral blood flow (CBF) with single-photon emission computed tomography (SPECT) in Alzheimer’s disease (AD) with reference to drug treatment (donepezil) and psychosocial intervention.

**Objective:**

The aim is to provide “brain-based” evidence for psychosocial interventions using SPECT.

**Methods:**

The participants were 27 consecutive outpatients with AD who received the drug and psychosocial intervention, and SPECT three times (baseline, pre-/post-intervention) at 6 month-intervals. The significance level of changes in CBF (Z score) and the extent of significantly changed areas, calculated with the eZIS system, were used as monitoring parameters. The participants were classified into three groups: improve (post-intervention CBF increased), worsening (progressive decline), and no change.

**Results:**

Six, 8, and 13 patients were classified as improve, worsening, and no change, respectively. All subjects in the improve group showed improvement in cognitive test scores for the MMSE and/or the CGI scores associated with the brain area with a CBF increase (right parietal lobe), suggesting appropriate psychosocial intervention (visuospatial intervention).

**Conclusion:**

These results suggest that monitoring of CBF with the eZIS system may be clinically applicable for monitoring of drug treatment and psychosocial intervention in AD patients.

## RESUMO

Nós examinamos previamente o fluxo sanguíneo cerebral (FSC) com tomografia computadorizada de emissão de fóton único (SPECT) na doença de Alzheimer (DA) com referência ao tratamento medicamentoso (donepezila) e intervenção psicossocial.

**Objetivo:**

Fornecer evidências “baseadas no cérebro” para intervenções psicossociais usando o SPECT.

**Métodos:**

Os participantes foram 27 pacientes ambulatoriais consecutivos com DA que receberam a droga e intervenção psicossocial, e SPECT por três vezes (basal, pré/pós-intervenção) em intervalos de seis meses. O nível de significância das mudanças no FSC (escore Z) e a extensão das áreas significativamente alteradas calculadas com o sistema eZIS foram utilizados como parâmetros de monitoramento. Os participantes foram classificados em três grupos: melhora (FSC pós-intervenção aumentada), piora (declínio progressivo) e nenhuma mudança.

**Resultados:**

Seis, oito e 13 pacientes foram classificados como melhora, piora e sem alteração, respectivamente. Todos no grupo melhora mostraram aumento dos escores no MEEM e/ou nos escores do CGI associados à área do cérebro com aumento do FSC (lobo parietal direito), sugerindo intervenção psicossocial apropriada (intervenção visoespacial).

**Conclusão:**

Estes resultados sugerem que o monitoramento do FSC com o sistema eZIS pode ser clinicamente aplicável para o monitoramento do tratamento medicamentoso e intervenção psicossocial em pacientes com DA.

Since no curative drugs are available for patients with dementia, especially Alzheimer’s disease (AD), psychosocial intervention is necessary for maintaining quality of life (QOL). Among the interventions, the reminiscence approach is usually performed based on the concept of “life review as a naturally occurring, universal mental process”.^1^ It consists of recollections and discussions of past events in the subject’s life with the aid of materials that remind them of their memories, and is classified into two methods: individual reminiscence and group reminiscence approaches (GRA). Reality orientation (RO)^2^ is another intervention aimed at reinforced recognition of orientation. Some researchers have reported^3^^-^ 7 that the GRA with RO exhibited synergistic effects, and a combined approach has been widely practiced.

In a survey^8^ of psychosocial interventions performed at Day Service Centers, we found that centers performed not only the GRA with RO but also collage, music activity, and physical activity.

Collage is a unique technique, constituting one of the well-known modern art approaches. It is sometimes used in psychosocial interventions for dementia patients as a part of creative and recreational activities.^9^ Holden et al.^10^ noted “these can be made in the group; members search through magazines looking for pictures that illustrate the particular theme, cut these out and stick them on a large piece of paper” (pg. 159-160). We previously recorded the themes in the collage articles shown by typical AD cases.^11^

For music activity, singing is a particularly effective means for patients with nonfluent aphasia or vascular dementia to produce words that they are unable to pronounce otherwise.^12^ Singing may facilitate speech at different stages of processing: at the motor stage by reducing the speech rate in patients with dysarthria,^13^ at the level of word retrieval by providing structural constraints, such as the number of syllables per beat,^14^ and at a motivational level by engaging recreational skills.

Regarding physical activity (PA) and dementia, the highest PA category is inversely associated with the risk of dementia and AD.^15^ PA has been reported to be a protective factor against incident dementia in a population-based cohort,^16^ although results remain controversial.^17^^,^ 18

Although the staff at Day Service Centers were found to perform according to their knowledge, brain-based background has not been fully investigated.

To reveal the neurological background of these psychosocial interventions without symptomatic drug treatment using cholinesterase inhibitors (ChEI), such as donepezil, only the GRA with RO was investigated. We recruited patients with vascular dementia (VaD) who were not indicated for the administration of such drugs. Using the fluorodeoxyglucose (FDG) method and positron emission tomography (PET), we noted^19^ that cerebral glucose metabolism in the anterior cingulate was increased after the GRA with RO together with an association of their increased activities of social interaction.

Using a more common method, SPECT and ^99m^Tc-ECD to examine the neurological background,^20^ we found that a combined therapeutic approach of GRA with RO and donepezil can directly stimulate the attention function and indirectly affect the judgment function based on observing other participants’ behaviors. Frontal cerebral blood flow (CBF) was higher in responders (as shown by MMSE scores), and parietal CBF was higher in those who recalled the contents of the intervention. Donepezil stimulated areas similar to those influenced by the intervention, and thus the drug was compatible with the intervention.

However, no such studies have been performed for psychosocial interventions other than the GRA with RO.

Application of neurological and neuroimaging findings is likely to improve the approach for treatment of dementia patients, and a scientific basis for psychosocial intervention may be provided based on neurological background. Herein, we report an observation study based on SPECT using ^99m^Tc-ECD. The aim is to provide “brain-based” evidence for psychosocial interventions.

## METHODS

This is an observational study, in consecutive outpatients with AD undergoing treatment with 5 mg/day of donepezil, and also participating in psychosocial interventions. The SPECT examination was performed 3 times (baseline, pre-intervention, and post-intervention) at 6-month intervals. The participants were clinically classified into 3 groups by a board-certified neuroradiologist independent of this study: improve (post-intervention CBF increased compared to pre-intervention CBF), worsening (progressive decline), and no change.

### Patients

We selected 27 subjects (10 men and 17 women, mean age 78.5 years) from consecutive outpatients with probable AD at the Dementia Clinic of Osaki-Tajiri SKIP Center, an integrated institute for medical care and welfare, using inclusion criteria of: 1) probable AD based on the NINCDS-ADRDA diagnostic criteria,^21^ 2) Mini-Mental State Examination (MMSE)^22^ score ≥10 for understanding instructions, 3) undergoing treatment with 5 mg/day of donepezil, and 4) participating in psychosocial interventions (see below); and exclusion criteria of: 1) concomitant cerebrovascular diseases shown by magnetic resonance imaging (MRI, see below), 2) apparent behavioral abnormalities such as delusion, agitation, or depression, and 3) decreased daily activities that could prevent participation in psychosocial interventions. For ChEIs, only donepezil was included since we could only use donepezil from 1999 to 2010 in Japan.

Written informed consent was obtained from all patients and from family members according to the Declaration of Helsinki. The study protocol was approved by the Ethical Committees of Tohoku University School of Medicine and the Osaki-Tajiri SKIP Center.

### Psychosocial intervention

Patients were asked to select which activities were preferred. Their care managers and families helped them with selection of the activities. They were all group activities performed once a week for more than 12 months. The facilitators were the staff at the Tajiri Fukushi-Kai (Welfare Association) and associated staff, their educational background being university graduates. The Tajiri Fukushi-Kai is run by Long-Term Care Insurance in Japan, and all the psychosocial interventions were performed in the Day Service or Day Care settings.

1. *Reminiscence approach with reality orientation:* There are four seasons in Japan, and the following festivals are traditionally held: *Oshogatsu* (New Year) in January, *Sestubun* (Bean Throwing Night) in February, *Hinamatsuri* (Girls’ Festival) in March, *Hanami* (cherry blossom party) in April, *Koinobori* (Boy’s Festival) and *Taue* (rice-planting) in May, *Tuyu* (rainy season) in June, *Hanabi* (fireworks) in July, *Bonodori* (Bon Festival dance) in August, *Higan* (autumn equinox) in September, *Inekari* (harvesting rice) in October, and *Toshikoshi* (New Year’s Eve) in December. The RO teaches the content of each festival and discusses the present situation. The GRA stimulates patients to access their remote memories of these festivals and to talk to each other about their memories.
2. *Collage technique as visuospatial activity:* As described earlier, collage is well known as a unique technique employed in modern art. It is sometimes used in psychosocial interventions for AD patients as a part of creative and recreational activities.^9^ Therapists have generally interpreted the expressions of individuals in terms of the framework of projective or symbolic theory. The participants were asked to use several pieces which were prepared by the staff, so as to create a “theme” for each session.
3. *Music activity:* As described earlier, singing is an effective means for patients with nonfluent aphasia to produce words that they are otherwise unable to pronounce.^12^^,^ 13 Singing may facilitate speech at different stages of processing: at the motor stage by reducing the speech rate in patients with dysarthria,^14^ at the level of word retrieval by providing structural constraints, such as the number of syllables per beat,^15^ and at a motivational level by engaging recreational skills. The participants were asked to engage in Karaoke for this activity.
4. *Physical activity:* Exercises included walking and step aerobics using *Stepwell 2* (Konami Sports & Life Co., Tokyo, Japan). The intensity of each set of exercise was adjusted to “13 (slightly hard)”, “14” or “15 (moderately hard)” using the Borg Rating of Perceived Exertion (RPE).

**Figure 1:**
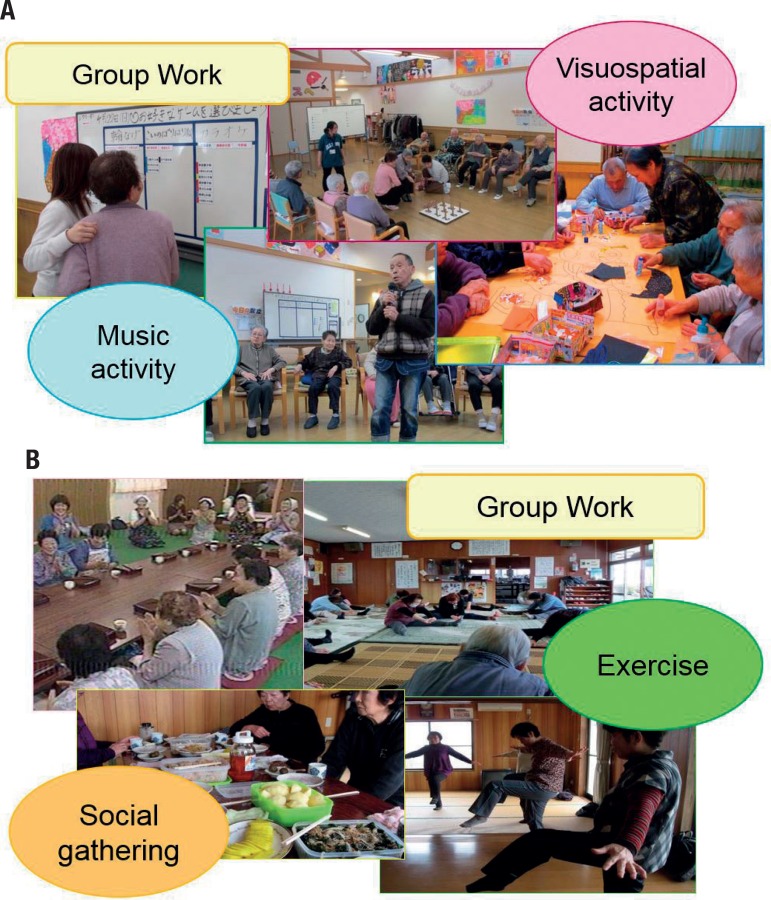
[A] music visuospatial activities [B] social gathering and exercise

Figure 1 Scenes of musical and visuospatial activities (a) and of a social gathering and exercise (b) in the community. A musical activity included Karaoke, a visuospatial activity involved collage and drawings. A social gathering included conversation over small dishes, and exercise involving stretching. All activities were performed once a week.

### MRI

A 1.5T-MRI system (Toshiba, Japan) was used to acquire T_1_-weighted images, T_2_-weighted images, and FLAIR images. CVDs were evaluated on axial and coronal images by a board-certified neuroradiologist who was blinded to other aspects of this study.

### SPECT

SPECT (Millennium MG, GE Yokogawa Medical Systems, Tokyo, Japan) was performed using the 99mTc-ethyl cysteinate dimer (99mTc-ECD). The details have been described elsewhere.^20^ The scan was performed 15 min after intravenous injection of 740 MBq of 99mTc-ECD. The acquisition protocol comprised 36 projections at 25 s per projection with 360° rotation of the camera. No quantitative analysis was performed for determination of CBF.

### Classification of patients

SPECT examination was performed 3 times (baseline, pre-intervention, and post-intervention) at 6 month-intervals at the same imaging center. Baseline SPECT images were used for confirming diagnosis, and after obtaining informed consent, pre-SPECT scanning was performed. Significant CBF changes (Z scores) and the extent of areas computed with the eZIS system were used as monitoring parameters. Based on the SPECT findings, the participants were classified into 3 groups: improve (post-intervention CBF increased compared to pre-intervention CBF), worsening (progressive decline), and no change.

### Cognitive assessment and clinical effect

We used the MMSE to assess global cognitive function and the Clinical Global Impression scale (CGI) (3 grades) to evaluate the clinician’s observation.^24^ All cognitive tests were performed by psychologists independent of the staff performing the psychosocial interventions and blinded to other results of the study. The CGI was also blindly assessed by a physician at the clinic. All cognitive assessments and the CGI were performed before starting the psychosocial interventions. The participants were also clinically classified into 3 groups: improve (post-intervention CBF increased compared to pre-intervention CBF), worsening (progressive decline), and no change.

### Analyses

Scoring was performed with the eZIS, developed by Matsuda et al.^23^ based on the same idea as SPM (eZIS is an automatic and free version of SPM in Matlab®). Using voxel-based spatial normalization, a SPECT image of a subject was converted into the same stereotaxic space as a normal age-matched database image. A voxel-wise z-score [{(CBF of patient)-(CBF of normal database)}/(SR)] was computed, and voxels above a suprathreshold (p<0.05) were detected as significant areas. The Chi-square test was used to analyze the relationship between the classification of participants and clinical changes.

## RESULTS

### CBF changes

**Figure 2:**
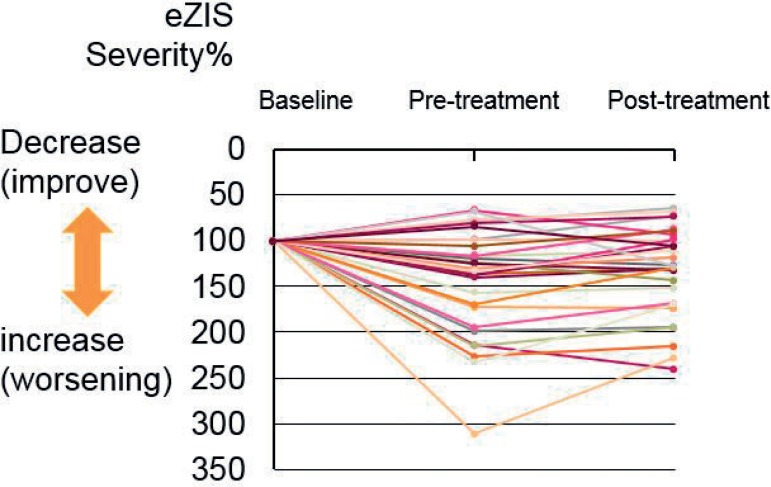
The eZIS sensitivity % changes for three SPECT examinations

The eZIS sensitivity % changes for three SPECT examinations of each patient are shown in Figure 2. Six, 8, and 13 participants were classified as improve (22.2%), worsening (29.6%), and no change (48.1%), respectively. All improve patients showed improvement of cognitive scores for MMSE and/or the CGI scores Seven of the 13 no change patients also showed clinical improvement.

### Case reports

For better understanding of the results, three typical cases are described below. The eZIS system colors areas in which CBFs are decreased compared with the normal database.

- Case TA, 82-year-old woman: visuospatial activity with donepezil (Figure 3)
  **Figure 3:**
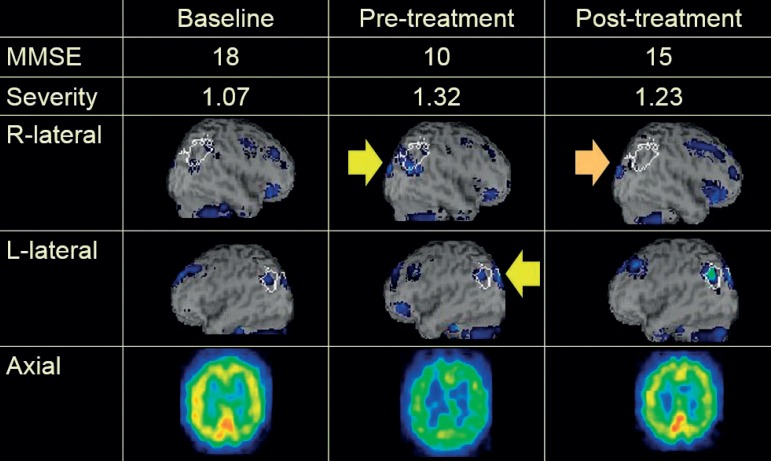
Visuospatial activity with donepezil
  After a comprehensive approach, CBF increased in the right parietotemporal area (post-treatment, colored areas decreased) compared with the left side. This suggests that the right parietotemporal area was stimulated by the treatment. The change in MMSE score shows that this patient was a good responder.
- Case FS, 86-year-old woman: music activity with donepezil (Figure 4)
  **Figure 4:**
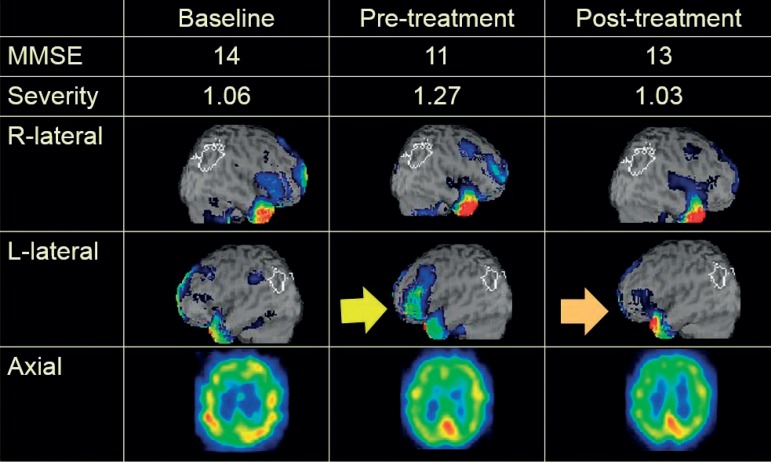
Music activity with donepezil
  A pre-treatment image showed a severe CBF decrease in the left lower frontal lobe (Broca’s area). After a comprehensive approach, CBF improved in this area, although the change in MMSE score was slight. This suggests that Broca’s area was stimulated by the treatment.
- Case MK, 83-year-old man: exercise with donepezil (Figure 5)
  **Figure 5:**
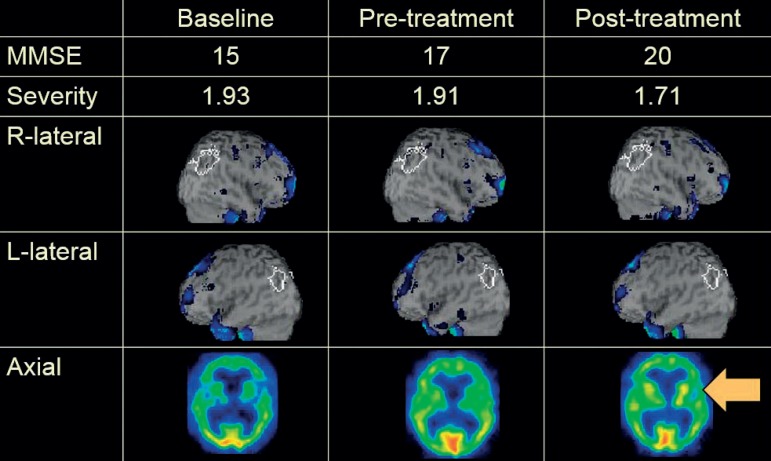
Exercise with donepezil
  A post-treatment image of a normal axial section illustrated increased CBF in the basal ganglia, although there were no marked changes on the eZIS images. This suggests that the basal ganglia was stimulated by the treatment. The change in MMSE score shows that this patient was a good responder.

### Relationship with clinical findings

Relationships between clinical findings and SPECT findings are shown in Table 1. A Chi-square test indicated that the findings were significantly correlated (sensitivity=0.89, specificity=0.20).

**Table 1:** Clinical improvement associated with SPECT findings.

		SPECT		
		Improve	No change	Worsening
**Clinical**	Improve	5	5	2
	No change	1	6	4
	Worsening	0	2	2

## DISCUSSION

The results of this study suggest that monitoring of CBF with the eZIS system using ECD-SPECT is clinically applicable for drug treatment and psychosocial intervention for AD patients. As described above, the neurological background has not been widely applied to psychosocial intervention by the staff at Day Service Centers. We believe that neurological and neuroimaging findings may facilitate a better approach to treatment of dementia patients by providing a scientific basis for psychosocial intervention. We hope the results can provide “brain-based” evidence for psychosocial interventions.

This is demonstrated by the neuroimaging findings in three typical cases. For Case TA, the right parietotemporal area was stimulated by donepezil treatment and visuospatial activity. The right parietotemporal area is associated with visuospatial function. Simplification and poor organization were also reported in previous studies on drawing impairments in AD,^25^^,^ 26 and these deficits are related to executive dysfunction in the frontal lobe and visuospatial dysfunction in the parietal lobe.

For Case FS, Broca’s area was stimulated by the drug plus music activity.

Clinically, some aphasic patients can sing well despite their speech disturbances. We previously reported^7^ that non-fluent aphasia patients complaining of difficulty finding words had improved speech function after singing training. All had lesions in the left basal ganglia or temporal lobe. They selected melodies they knew well, but which they could not sing, and we produced new lyrics with a familiar melody using words that they could not identify. Patients with an intact right basal ganglia and left temporal lobes, together with preserved right hemispheric glucose metabolism on positron emission tomography, might be candidates for effective singing therapy.

For Case MK, the basal ganglia was stimulated by the drug with exercise. Physical activity has been shown to diminish age-related brain volume shrinkage in several brain regions, accompanied by a reduction of age-related decline in cognitive functions. Niemann et al.^27^ revealed that motor fitness was positively associated with the volume of the basal ganglia, and provided evidence that coordinative exercise is a favorable leisure activity for older adults with the potential to improve the volume of the basal ganglia. The basal ganglia is also associated with pathophysiological conditions such as Parkinson disease.^28^ The same influences may underlie AD patients.

We should mention several limitations. This is a retrospective, observational study without controls. Strictly speaking, patients who received donepezil without psychosocial intervention should have been used as controls. However, the staff at the Osaki-Tajiri SKIP Center first consider life support for dementia patients, and any kind of psychosocial intervention is provided prior to drug treatment; thus, it is practically difficult to establish controls. Also, more reliable results would be obtained from a prospective study with random classification of the patients into each psychosocial intervention. However, this is also practically difficult since the interventions were chosen by the patients based on their favorite activities. Also, we cannot know whether the findings will persist after stopping the intervention. Despite these limitations, the current findings provide clinically important information on the effects of interventions on neurological background.
